# Comparison of two methods of cardiopulmonary exercise testing for assessing physical fitness in children and adolescents with extreme obesity

**DOI:** 10.1007/s00431-022-04434-7

**Published:** 2022-03-12

**Authors:** Linda Kalski, Martin Wannack, Susanna Wiegand, Bernd Wolfarth

**Affiliations:** 1 Institute of Sport Science, Humboldt-Universität zu Berlin, Berlin, Germany; 2 Department of Sports Medicine, Charité – Universitätsmedizin Berlin, Berlin, Germany; 3 Center for Social-Pediatric Care/Pediatric Endocrinology and Diabetology, Charité – Universitätsmedizin Berlin, Berlin, Germany

**Keywords:** Obesity in children and adolescents, Cardiopulmonary exercise testing, Bicycle, Treadmill

## Abstract

It is well-known that children and adolescents with obesity have increased over recent decades which in turn carries greater risk of co-morbidities and poses a preventive as well as a therapeutic challenge. Currently, there are limited recommendations available on proven methods for recording physical fitness in children and adolescents presenting with extreme obesity. In this study, twenty participants, aged 12–17 years, with a body mass index (BMI) above the 99.5th percentile, were comparatively assessed, using a correlation between their physical fitness on a bicycle (BC) and treadmill (TM) cardiopulmonary exercise testing (CPET) with a lactate diagnostic. The results of the BC and the TM were as follows: maximum heart rate (HR_max_) 186.4 ± 8.6 beats per minute (bpm) vs. 190.8 ± 8.8 bpm, peak oxygen consumption (VO_2_peak/kg) 23.5 ± 2.9 ml/min/kg vs. 25.4 ± 3.1 ml/min/kg, and maximum lactate (La_max_) 6.4 ± 1.6 mmol/l vs. 5.6 ± 1.4 mmol/l. The values of HR_max_ and VO_2_peak/kg were significantly higher for adolescents tested on the TM. However, no significant difference was observed in either La_max_ values or between the genders.

*Conclusions*: The higher values of HR_max_ and VO_2_peak/kg could be attributed to the activation of a higher percentage of muscle mass on the TM. Lower La_max_ values on the TM suggest maximum physical exertion was not achieved. This could be due to the extreme body weight carried by the participants. Both the BC and the TM CPET could be used for assessing physical fitness in children and adolescents with extreme obesity but should not be used interchangeably.

**Table:** 

**What is Known:** *• Currently, there are only limited recommendations available on proven methods for recording physical fitness in children and adolescents with extreme obesity available.*	
**What is New:** *• Cardiopulmonary exercise testing with maximum physical exertion has been shown to be feasible in children and adolescents with extreme obesity. The results obtained from this study demonstrated that both a bicycle and a treadmill can be effectively used for assessing the physical fitness levels in children and adolescents with extreme obesity.*

**What is Known:**

*• Currently, there are only limited recommendations available on proven methods for recording physical fitness in children and adolescents with extreme obesity available.*

**What is New:**

*• Cardiopulmonary exercise testing with maximum physical exertion has been shown to be feasible in children and adolescents with extreme obesity. The results obtained from this study demonstrated that both a bicycle and a treadmill can be effectively used for assessing the physical fitness levels in children and adolescents with extreme obesity.*

## Introduction

Obesity in childhood and adolescence is a growing and worldwide issue [1]. The KiGGS study, a representative population-based survey [2], showed that, in Germany, 15.4% of the adolescents are overweight and 5.9% are obese [3]. Extreme obesity is defined by a body mass index (BMI) above the 99.5th percentile. This corresponds to a standard deviation score (SDS) of more than + 2.5. Extreme obesity in childhood and adolescence is associated with an increased metabolic and cardiovascular morbidity (e.g., type 2 diabetes or coronary heart disease) in adulthood [4].

Physical fitness encompasses both physical and psychological components, although sports science only takes the physical components into consideration. According to Weineck [5], physical fitness defines the degree of expression of a certain sports motor performance. Physical fitness can be determined and assessed during maximal exercise testing. Using standardized examination conditions, performance diagnostics can be conducted on an ergometer. Evaluation of the results enables athletes, as well as recreational athletes and study participants, to receive specific training recommendations, based on findings. Additionally, results can provide supplementary therapy planning for people who are suffering from different diseases [6].

Physical activity plays an important role in prevention and treatment of overweight and obesity in childhood and adolescence [7]. Indeed, several recent publications have put forward different types of physical activities that have been shown to be effective in fighting obesity [8–10]. Dias et al. [10] have demonstrated how high-intensity interval training can be effective for people with obesity, especially by decreasing BMI-SDS and increasing cardiorespiratory fitness. For correct execution and highest levels of effectiveness, high-intensity interval training needs to be performed at a high-level heart rate. However, there is currently very little data on adolescents with extreme obesity exercising at such high-level heart rates. Hansen et al. [11] found in their systematic review and meta-analysis that there are differences between obese and lean adolescents in VO2peak measurements. Klijn et al. [12] found relevant and significant improvements on aerobic performance in adolescents with extreme obesity following an aerobic training program. When treating children and adolescents with extreme obesity, it is essential to consider individual performance limits and to make recommendations based on the data.

The aim of the present study was therefore to compare two methods of cardiopulmonary exercise testing (CPET) in terms of their feasibility and efficiency for maximum physical exertion in children and adolescents with extreme obesity. Additionally, possible gender-specific differences in physical fitness were also considered. Analysis of each individual’s physical fitness could, potentially, in addition substantiate personalized therapy recommendations already in place.

## Methods

Written informed consent was obtained from each participant prior to testing. All examinations were carried out at the outpatient clinic of the Department of Sports Medicine at Charité – Universitätsmedizin Berlin/Humboldt, University of Berlin. Participants were recruited from the Center for Social-Paediatric Care of the Charité Berlin.

### Participants

All participants were free of any medical condition that could have affected their performance of medical measurements. Exclusion criteria were individuals under 10 or over 17 years of age and body weight < 97. Percentile or BMI is < 25. The participants (*n* = 20; f: *n* = 11; m: *n* = 9) were 12 to 17 years of age (15.2 ± 1.6) at the time of examination. Table 1 shows anthropometric data of the study population with mean values and standard deviation for age, BMI, BMI-SDS, body fat percentage, and achieved power of each CPET.

**Table 1:** Anthropometric data

**Sex**	***n***	**Age [years]**	**Height [cm]**	**Weight [kg]**	**BMI [kg/m**^2^**]**	**BMI-SDS**	**BFP [%]**	**BFM [kg]**	**FFM [kg]**	**Power, relatively BC [W/kg]**	**Power, absolutely TM [%]**
**Male**	9	14.3 (± 1.5)	174.5 (± 10.0)	112.1 (± 26.1)	36.4 (± 4.8)	2.9 (± 0.4)	41.4 (± 6.7)	46.5 (± 13.7)	65.8 (± 15.3)	1.2 (± 0.2)	17.6 (± 2.3)
**Female**	11	15.8 (± 1.4)	168.6 (± 8.4)	102.7 (± 20.1)	36.1 (± 6.0)	3.1 (± 0.7)	45.0 (± 4.8)	46.8 (± 12.2)	56.3 (± 9.0)	1.2 (± 0.3)	16.6 (± 2.6)
**All**	20	15.2 (± 1.6)	171.3 (± 9.1)	106.9 (± 22.4)	36.2 (± 5.5)	3.0 (± 0.6)	43.4 (± 6.0)	46.7 (± 12.9)	60.6 (± 13.1)	1.2 (± 0.3)	17.0 (± 2.5)

Data of participants described as mean values and standard deviation in brackets

*n* number of participants, *BMI* body mass index, *BMI-SDS* standard deviation score for body mass index, *BFP* body fat percentage, *BFM* body fat mass, *BC* bicycle, *TM* treadmill, *FFM* fat-free mass

Only subjects who participated in both examinations (BC and TM) were included in this study. There is, therefore, no missing data in this record.

### Study design

Participants were tested in an incremental bicycle ergometry and running test in a laboratory setting (see protocols below) on two different days within a time frame of 2 weeks. In the first week, the bicycle exercise test was performed with the treadmill exercise test being carried out 1 week later. The tests were not carried out under fasting conditions, as this is not recommended for maximal exercise testing. Data collection started in July 2018 and finished in January 2019. Due to the pilot trial, a sample size calculation was not necessary.

### Anthropometric data

The following anthropometric data were measured on two different days: body height, body weight, waist-to-hip ratio (WHR), BMI, caliperometry, and body composition with bioelectrical impedance analysis. The average of each data was subsequently calculated.

With the participant in an upright position, the body height and body weight of each were measured using a calibrated scale (Seca 285 DP, Seca GmbH & Co. KG, Hamburg, Germany). To calculate the BMI of each subject, the formula *BMI* = kg/m2 was used. Subsequently, waist and hip circumference was measured in cm with a standardized tape measure (Seca GmbH) in cm to determine the WHR. The WHR is the dimensionless ratio of the circumference of the waist to that of the hips and is calculated as waist measurement divided by hip measurement.

The anthropometry of fatty and fat-free mass was based on caliperometry of 7 and 10 skin folds. First, the thickness of designated skin folds was obtained using a caliper (Harpenden Skinfold Caliper, UK). A total of eleven different points of the body were included in the measurement, allowing the percentage body fat to be calculated using formulas from Jackson and Pollock and Johnson and Scholz [13–15]. Additionally, a bioelectrical impedance analysis was repeated at each appointment to measure body composition before exercise testing, to allow changes to be recorded. A body analysis scale (InBody770, JP Global Markets GmbH, Eschborn, Germany) was used to determine muscle, fat, and water proportions by measuring resistance [16, 17].

### Exercise testing

Prior to the test, participants were informed of the importance of maximal physical effort. They were, however, instructed to immediately report any occurrence of thoracic pain, limiting dyspnea, or dizziness experience during testing. Prior to the beginning of each exercise, a clinical examination and an inconspicuous 12-lead rest electrocardiogram (ECG) (Custo Cardio 200 Saug-EKG, Custo med GmbH, Ottobrunn, Germany) were mandatory. During the test, a 12-lead ECG (Custo Cardio 300) was continuously running and monitoring (Software Custo diagnostics, Version 4.6.4).

After completion of the BC test, the running examination on the TM was planned after a 1-week break. This sequence was chosen deliberately, as experience has shown that the ECG quality during BC testing is improved, and any abnormalities can be detected at an earlier stage. Both examination methods were conducted under standardized laboratory conditions. The ambient temperature was between 18 and 24 °C with a humidity of 30–60%.

All participants were encouraged by the medical staff to exercise at their maximal effort level. During the test, the subjective state and the rating of perceived exertion (RPE) were recorded by the BORG 20 scale (Borg RPE) [18, 19]. The end of the test was generally determined by the participants’ maximum exhaustion.

### Bicycle ergometer (BC)

Adjustments were made to the height of the BC saddle (Ergoselect 100 k. Ergoline GmbH, Bitz, Germany) and handlebars prior to testing. After a rest period of 3 min, the participants started at 25 W with a 25 W increase every 3 min. The ideal pedaling cadence was a consistent speed of 60–70 RPM, and no less than 60 RPM. At the point of exertion, the test was concluded, and participants remained on the BC at a low frequency with light resistance so that there was no abrupt load termination.

Throughout testing, ECG recordings continued to monitor recovery, ensuring 5-min rest. During the test, the blood pressure was measured every 3 min, with a standard suitable arm cuff, and a blood sample from the earlobe was taken every 3 min for lactate diagnostic purposes.

### Treadmill ergometer (TM)

A constant speed on the TM (h/p cosmos sports & medical GmbH, Nussdorf-Traunstein, Germany) was set at 4 km/h with an initial elevation of 2.5%. This was increased by 2.5% every 3 min until the participant was seen to be exhausted. The protocol was selected [20] and needed to be adjusted for each participant, because high-speed running is not recommended for people with extreme obesity. The intensity of the exercise was therefore increased by raising the elevation rather than the speed of the TM.

Between the 3-min training intervals, a 30-s rest was given to measure lactate accumulation (LA) from capillary blood samples taken from the earlobe. Blood pressure was measured at the beginning of the test, at the point of exertion (until the end of the test), and after a minimum of 5 min in a resting position. The ECG was recorded continuously as in the first test on BC. The participants were able to stop the examination at any time.

### Lactate accumulation (LA)

In order to be able to determine the blood lactate concentration, capillary blood was taken from the earlobe [21]. The earlobe had already been prepared with a blood flow-enhancing warming ointment (Finalgon) and then disinfected. LA was taken once at rest, during the test in the last 10 s of each stage and 3 times during the recovery phase. Immediately after the recovery phase, the blood samples were placed in a sample tray of the lactate device EKF Diagnostics Biosen C-line Clinic (Barleben, Germany) and analyzed within a few seconds by enzymatic-amperometric chip-sensor technology. Incremental exercise tests eliciting an increase in lactate values were then used to produce lactate curves. The lactate curve could be used to determine the aerobic and anaerobic threshold and may serve as a basis for individually assessing endurance capacity and training zones. In addition, a maximum lactate concentration provided proof of maximum exertion [22].

### Cardiopulmonary exercise testing (CPET)

After a spirometry, a bodyplethysmography was conducted for each participant. Both resting pulmonary function tests were performed using COSMED Q-Box with COSMED Quark PFT (Pavona di Albano, Italy). This procedure made it possible to diagnose respiratory impairment prior to CPET [20].

Noninvasive metabolic testing equipment (K5 Wearable Metabolic System, COSMED, Pavona di Albano, Italy) supplied comprehensive measurements and therefore invaluable information about physical fitness and individual exercise limits, from which the training status could be generated [23].

### Self-reported questionnaire

In order to gain further insights in the subjects’ well-being during both CPET, all subjects received a short questionnaire at the end of the second examination. Each of the subjects filled out the questionnaire themselves, providing subjective feelings of perceived exertion as a supplement to the BORG RPE scale. The combination of both tools was used so that the subjects did not have any difficulty in assessing their subjective perception of effort during exercise. Six age-appropriate questions were asked on parameters such as effort, joy, comfort, discomfort, motivation, and ambition, and the subjects could each choose a preferred CPET.

### Data evaluation/statistics

The collected anthropometry data were recorded using a clinic software *[I/med]* (Version 6.34, DORNER GmbH & Co. KG). Resting ECG and stress ECG were recorded using Custo diagnostics (Custo med GmbH Version 4.6.4). The results of both pulmonary function tests and CPET were obtained using Omnia software from COSMED 2013/06). The data of performance diagnostics and in particular the LA values were visualized and recorded in Ergonizer (Version 5.0.1 Build 134; Röcker, 2013), and all data were transferred into an Excel spreadsheet (Microsoft Office Excel, 2019). Data were processed with the statistical program SPSS (IBM, SPSS Statistics, Version 25). For descriptive statistics, i.e., anthropometric data, respective mean values and standard deviations (SD) were calculated using SPSS.

A significance level of *p* < 0.05 was selected in the following evaluation to determine statistical significance [24]. Using SPSS, an analysis of variance (ANOVA) with repeated measures is applied as part of the inferential statistics. First, the residuals were tested for assumption of normality. The homogeneity of variance was subsequently proved, so that an ANOVA could be given [25]. ANOVA was performed on three parameters (HR_max_, La_max_, and VO_2_peak/kg) and calculated for the effects of ergometry (within-subject factor, i.e., intraindividual) and sample and supplemented with the variable gender as a between-subject factor, i.e., inter-individual [24]. For the scatterplots, the software R (version 4.0.4) with the R-package ggplot2 (version 3.3.3) was used.

## Results

### Power

The participants achieved a mean power of 160.4 ± 6 W on BC. The girls reached an average of 155.6 ± 37 W and the boys 166.3 ± 23.6 W. As shown in Table 1, the boys reached a relative power of 1.2 ± 0.2 W/kg on the BC and the girls 1.2 ± 0.3 W/kg. In relation to fat-free mass, the subjects had a relative power of 2.7 ± 0.4 W/kg FFM. The boys reached a relative power of 2.5 ± 0.3 W/kg FFM, and the girls reached a relative power of 2.8 ± 0.4 W/kg FFM. On average, the participants achieved a 17.1 ± 3.4% slope at a constant speed of 4 km/h on the TM. The average load for the girls occurred at a mean slope of 16.6 ± 2.6%, and the boys achieved a 17.6 ± 2.3% slope.

### Maximum heart rate (HR_max_)

The achieved HR_max_ was on average 186.4 ± 8.6 bpm during BC testing and hence slightly lower than for TM testing which was 190.8 ± 8.8 bpm. For the female participants, the HR_max_ was 186.7 ± 8.1 bpm on BC and 190.9 ± 6.8 bpm on the TM. For the male participants, the HR_max_ on the BC was 186 ± 9.7 bpm and 190.6 ± 11.2 bpm on the TM.

### Maximum lactate (La_max_)

The La_max_ average was 6.4 ± 1.6 mmol/l on the BC and 5.6 ± 1.4 mmol/l on the TM. Female participants obtained a La_max_ of 6.3 ± 1.7 mmol/l on the BC and 5.5 ± 1.2 mmol/l on the TM. Male participants achieved a La_max_ of 6.5 ± 1.5 mmol/l on the BC and 6.0 ± 1.8 mmol/l on the TM.

### Absolute and relative VO_2_peak

The absolute VO_2_peak for all subjects was, on average, 2.5 ± 0.4 l/min on the BC and 2.7 ± 0.5 l/min on the TM. The female subjects reached 2.4 ± 0.4 l/min on BC and 2.6 ± 0.5 l/min on the TM. Male subjects achieved 2.6 ± 0.4 l/min on the BC and 2.9 ± 0.5 l/min on the TM.

The achieved relative VO_2_peak/kg was on average 23.5 ± 2.9 ml/min/kg on the BC CPET and 25.4 ± 3.1 ml/min/kg on the TM. The female participants reached 23.3 ± 2.7 ml/min/kg on BC and 24.9 ± 3.4 ml/min/kg on the TM. Male participants obtained 23.7 ± 3.2 ml/min/kg on the BC and 26.0 ± 2.8 ml/min/kg on the TM.

### Self-reported questionnaire

The two methods of CPET were compared on subjective parameters taken from the questionnaire. Fifteen subjects wrote that the cycling was more enjoyable than walking (five). In this context, seventeen subjects also felt more comfortable and safe on the BC, whereas only three subjects preferred the TM. Thirteen subjects reported being more ambitious and motivated when cycling than walking (seven). In terms of effort, the TM got more votes (twelve) than the BC (eight).

## Discussion

Children and adolescents with extreme obesity are able to achieve maximum physical exertion on CPET. We demonstrated that both the BC and the TM are suitable for CPET for this specific age and weight group. The advantages of using a BC are a more accurate evaluation of the exercise ECG and a more comfortable measurement of blood pressure [26]. A sitting position is considered safer and more comfortable, especially for obese patients. A conscious decision was made to use this device because a higher muscular and therefore cardiovascular exertion could be achieved on a standard bicycle ergometer than on a recumbent ergometer. A self-reported questionnaire revealed that the BC was more comfortable and probably more familiar than the TM. This also led to the assumption that the examination carried out on the TM was perceived as more strenuous for the subjects. It is important to mention that the questionnaire is not a valid instrument but was used in addition to the BORG RPE scale. On the other hand, the TM examination led to significantly higher levels of HR and VO_2_peak/kg. This could be considered as a better achievement of a maximum physical exertion [27, 28]. Another explanation could be that due to the high body weight, exhaustion and an anaerobic metabolic state were reached more quickly, and thus, the maximum load is reached sooner. This could result in a higher percentage of activated muscle mass. The subjects may have been unable to reach maximum muscular exertion due to reaching the cardiopulmonary maximum on the BC [23, 29].

Criteria for maximum physical load (and their most common definitions) are as follows: (I) plateau of VO_2_ (with an increase of less than 150 ml/min), (II) HR_max_ (+ / − 10 bpm of age-dependent HR_max_), (III) respiratory quotient RQ (VCO_2_/VO_2_ > 1.10), (IV) La_max_ (> 8 mmol/l), and (V) Borg RPE scale (> 18/20) [30]. These criteria are used in a standardized way for athletes and helped as a reliable orientation in this study [31]. The participants achieved maximum physical load by obtaining a Borg RPE scale value of at least 18 or more. The mean RQ of our participants was, on the BC 1.08 + / − 0.1 and 1.09 + / − 0.1 on the TM. Due to the lack of regular physical training and low exercise level of our participants, these two criteria were fulfilled. The previous study has found a lowered RERpeak in obese adolescents in contrast to lean adolescents [32]. Therefore, the present study has proved that both CPETs can determine of maximum physical load in children and adolescents with extreme obesity. Recent studies have shown that a supramaximal validation test to confirm VO2max is unaffected by sex, cardiorespiratory fitness level, and especially body mass [33]. Regarding the maximum effort evaluation, these supramaximal validation tests could be suitable for children and adolescents with extreme obesity.

The main limitation of the present study was the modest number of participants due to the study being explorative in nature. Based on the inclusion criteria, only a few subjects were eligible. The main issue with participant acquisition was the necessity for a two-staged examination over a 1- to 2-week period. Forty-five families were informed and received information sheets. Twenty-five of these families did not take up the opportunity. Additionally, both the BC and the TM represented a very unusual activity situation for the participants, so that the subjective rating of maximum exertion (Borg RPE scale) was probably reached before maximum cardiopulmonary load. One implication of the study was that the examinations on the TM led to a higher level of motivation at the second appointment, because the participants were eager to improve their previous achievements [34]. Regarding the significantly higher levels of HR_max_ and VO_2_peak/kg in TM, this CPET should be considered as the more valid one for cardiopulmonary performance assessment. This is in line with findings in several other studies [27, 28, 35]. In this study, participants showed lower levels of La_max_ on the TM. These values (*p* = 0.076), however, did not reach a significance level of *p* < 0.05. A lower La_max_ in the TM compared to the BC could be due to a higher demand for lactate as a source of energy by both cardiac muscle fibers and the active muscles that have no heavy involvement in propulsion, f.e. arms and shoulder muscles [36–38]. Further studies comparing both methods need to be performed to analyze whether this is due to a lower level of fitness in children and adolescents with extreme obesity or whether other TM protocols need to be applied.

Interestingly, there were no significant differences between male and female participants, which comparable results for HR_max_, La_max_, and VO_2_peak/kg in both male and female participants. There are usually differences in gender-specific athletic performances [39]. These results can be explained by the homogeneity of the participants. Both, females and males, were most likely unfamiliar with this kind of physical activity and reached a considerably lower maximum power on BC performed by normal weight boys (3–4 W/kg) and girls (2.8–3.5 W/kg) [40].

**Fig. 1:**
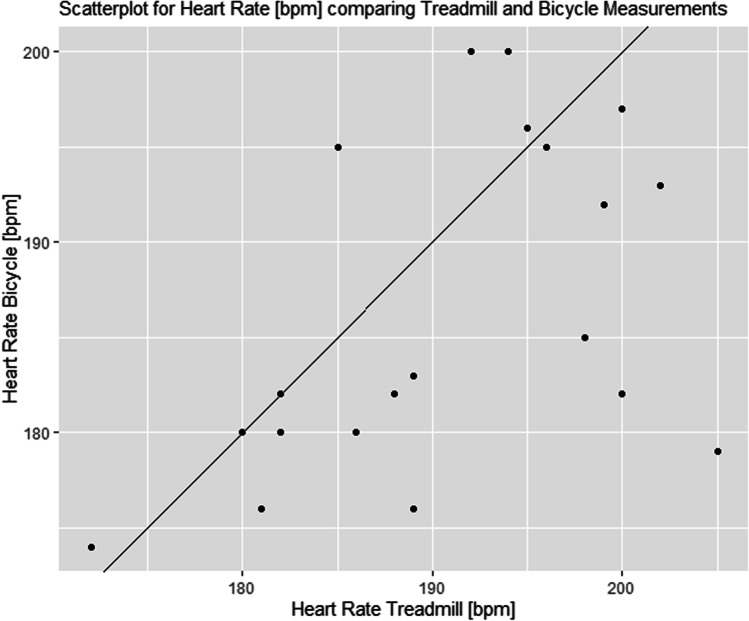
Differences in maximum heart rate between the treadmill and the bicycle compared to average measurements of each subject. The black line refers to a mean to the mean difference of 4.4 beats per minute. Consequently, using an ANOVA, a statistically significant effect was calculated for the within-subject factor method (cardiopulmonary exercise testing: bicycle and treadmill), *F* = 4.848, *p* = 0.041; i.e., *p* < 0.05. There was no significant effect for the interaction method × sex (*F* = 0.005, *p* = 0.945; i.e., *p* > 0.05)

**Fig. 2:**
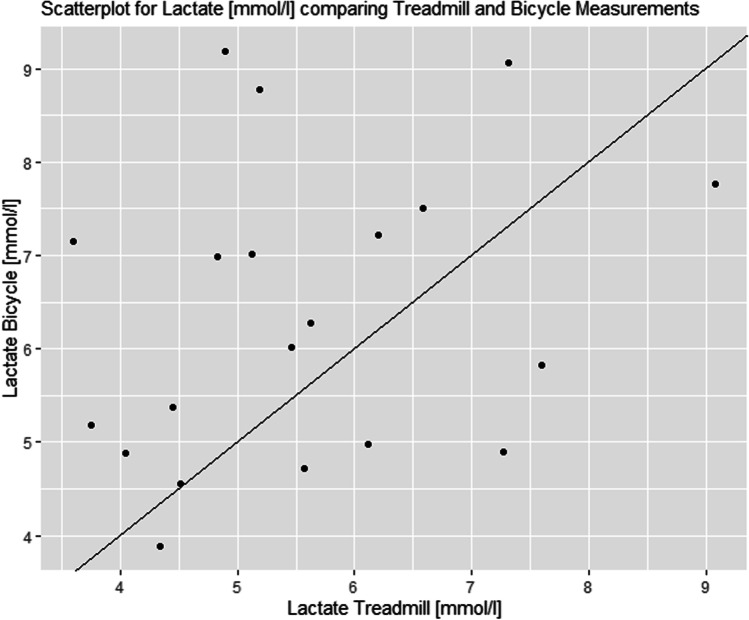
Differences in maximum lactate between the treadmill and the bicycle compared to average measurements of each subject. The black line refers to a mean difference of − 0.79 mmol/l. Using an ANOVA, which did not show significant differences for the within-subject factor method (cardiopulmonary exercise testing: bicycle and treadmill), *F* = 3.558, *p* = 0.076; i.e., *p* > 0.05. There was also no significant effect with the interaction method × sex (*F* = 0.000, *p* = 0.983; i.e., *p* > 0.05)

**Fig. 3:**
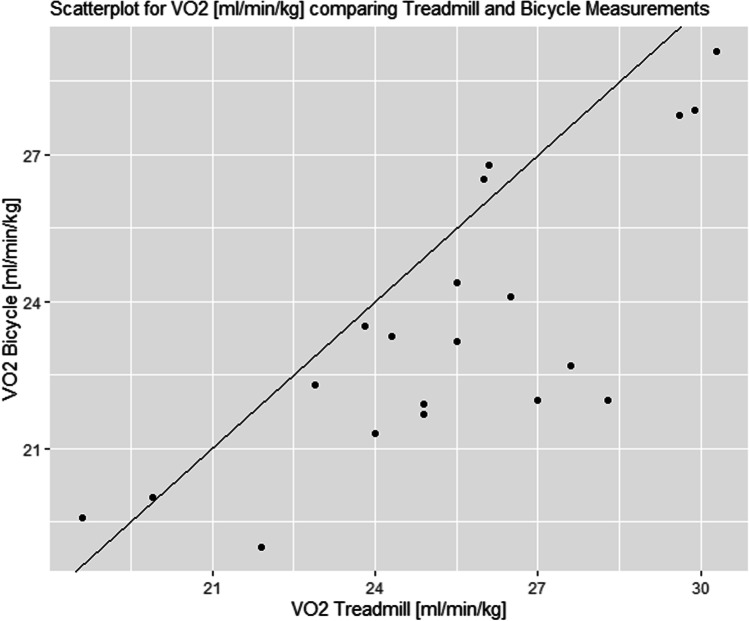
Differences in relative VO_2_peak between the treadmill and the bicycle compared to average measurements of each subject. The black line refers to a mean difference of 1.92 ml/min/kg. Using an ANOVA, a statistically significant effect was calculated for the within-subject factor method (cardiopulmonary exercise testing: bicycle and treadmill), *F* = 19.049, *p* = 0.000; i.e., *p* < 0.01. There was no significant effect with the interaction method × sex (*F* = 0.630, *p* = 0.438; i.e., *p* > 0.05)

Given the fact that the BC and the TM are easily accessible instruments for measuring the maximum physical load in children and adolescents with extreme obesity, both methods could potentially be used as longitudinal parameters in intervention studies. Further studies must be performed to optimize the exercise protocols, especially the TM protocol (Figs. 1, 2 and 3).

## Data Availability

All data and materials support our published claims and comply with field standards.

## Abbreviations

ANOVA: Analysis of variance
BC: Bicycle ergometer
BMI: Body mass index
CPET: Cardiopulmonary exercise testing
ECG: Electrocardiogram
HR: Heart rate
LA: Lactate accumulation
RPE: Rating of perceived exertion
SDS: Standard deviation score
TM: Treadmill ergometer
VO_2_peak: Peak oxygen consumption
WHR: Waist-to-hip ratio
